# Dissipative, Entropy Production Systems across Condensed Matter and Interdisciplinary Classical vs. Quantum Physics

**DOI:** 10.3390/e24081094

**Published:** 2022-08-09

**Authors:** Adam Gadomski

**Affiliations:** Faculty of Chemical Technology and Engineering, Institute of Mathematics and Physics, Group of Modeling of Physicochemical Processes, Bydgoszcz University of Science and Technology, 85-796 Bydgoszcz, Poland; agad@pbs.edu.pl

This Special Issue collected ten papers addressing a range of topics in condensed matter and interdisciplinary classical vs. quantum physics. All of them are linked together by statistical physics/mechanics methods. The selection emerged from a survey of topics presented at the XLVII Congress of Polish Physicists held in Bydgoszcz, Poland on the 19–23 September 2021 (http://47zfp.utp.edu.pl/en/47th-congress-of-the-polish-physical-society/ (accessed on 4 August 2022)). The topics address problems of classical (CL) and quantum (QU) statistical physics. As for the CL side, these comprise: multistability and ergodicity breaking; the formation of dissipative (e.g., spherulitic) structures in a nonequilibrium bath; ion-influenced conformation of biopolymers (glycosaminoglycans) near biosurfaces; infectious-disease propagation in terms of social distancing; far-from-equilibrium information processing employing networks of chemical oscillators; and a problem of equivalence of the Carnot principle to the second law of thermodynamics, and non-equivalence to the first Clausius and Kelvin principles. As for the QU side, in turn, these include: mixedness, coherence and entanglement in three-qubit states; (anti)coherence caused by a thermal bath; and finally, quantum graphs split at vertices as simulated experimentally by using microwave networks. All contributions can be united under the common flagship of maximum-entropy and entropy production principles experienced by the respective CL and QU systems in (non)equilibrium conditions.

The XLVII Congress of Polish Physicists was, for the first time in over hundred years in the history of the Polish Physical Society, held in Bydgoszcz, Poland on the 19–23 September 2021 (http://47zfp.utp.edu.pl/en/47th-congress-of-the-polish-physical-society/ (accessed on 4 August 2022)) as a hybrid event, gathering in total more than two hundred fifty regular participants and more than forty invited speakers. Amongst them, one may find the name of a Nobel Prize winner (2004), Professor Frank Wilczek, with his special online contribution titled “Time Crystals”. Over the course of five days and around fifteen sessions, covering topics ranging from physics’ teaching and popularization, the history of physics, and general physics to the physics of high energy and cosmology, gravitation, and astrophysics, the participants presented their recent contributions to the field. One of those sessions was titled Statistical, Nonlinear and Complex Systems’ Physics, whereas the other one, relevant for this Special Issue (SI), was the session titled Condensed Matter Physics. (Two other sessions on quantum information and photonics, as well as on biological and interdisciplinary physics, complete the selection of valuable contributions to this SI.) However, from these two sessions, one may expect to have a topical reflection contained in the overarching SI.

The thematic range of the selected contributions seems to be broad and can loosely be described as polydispersive. For example, it resembles a polynuclear path of yielding (poly)crystals. It can be so taken when looking at it from the first side. However, a closer inspection of the SI material gives rise to a much more monodispersive/single-crystal and compacted (than crudely expected) picture of the SI contents presented to a potential reader. Namely, all contributions collected can be united under the common denominator of maximum-entropy and entropy production principles experienced by both classical (CL) and quantum (QU) systems in (non)equilibrium conditions. In what follows, let us unveil the main messages conveyed by the ten collected papers of the SI. The proposed order of presenting the material commences with properly subordinated CL systems (seven contributions) and ends up with three remaining QU systems, presented by their authors.

The paper “Velocity Multistability vs. Ergodicity Breaking in a Biased Periodic Potential” [1] investigates the concept of multistability, i.e., the coexistence of several attractors for a given set of system parameters—it is one of the most important phenomena occurring in dynamical systems. The authors consider it in the velocity dynamics of a Brownian particle, driven by thermal fluctuations and moving in biased periodic-potential conditions. At non-zero temperatures, the ergodicity is roughly restored, whereas in low-temperature conditions, as occur in chaotic systems, the velocity dynamics are continuously affected by initial conditions due to weak ergodicity breaking. For moderate and high temperatures, the multistability is preserved with respect to the choice of both the starting position and velocity of the Brownian particle.

In the paper titled “Accumulation of Particles and Formation of a Dissipative Structure in a Nonequilibrium Bath” [2] it is demonstrated that in the non-Gaussian (Lévy noise) conditions, an inhomogeneous distribution of particles can be seen as a dissipative structure, i.e., a lower-entropy steady state of a system that permits the throughput of energy and the concurrent production of entropy. After the mechanism that maintains nonequilibrium is switched off, the relaxation stepping back to homogeneity gives rise to an increase in entropy and a corresponding decrease in free energy. Intuitive analytical formulae are proposed to connect the nonequilibrium Lévy noise and active particle behavior to the entropy decrease and energy increase.

In the study “Spherulites: How Do They Emerge at an Onset of Nonequilibrium Kinetic-Thermodynamic and Structural Singularity Addressing Conditions?” [3], the authors discuss, within a non-dimensional physical–kinetic model for far-from-equilibrium growth, the formation of special polycrystalline objects, named spherulites, exhibiting different growing-mode conditions. It appears that according to the modeling offered, it is feasible to foresee that the spherulites’ emergence is possible prior to a pure diffusion-controlled (poly)crystal growth. It was demonstrated that the spherulitic onset addressing factors of the two different evolution types of spherulitic growth modes, namely, diffusion-controlled growth and mass-convection-controlled growth, emerge from the modeling performed. This resulted in accepting a conclusion that a type of liaison of amorphous and crystalline phases makes the system far better compromised to the actual (sometimes, concurrent) thermodynamic–kinetic conditions.

The proposal of a prerequisite of a (paradigmatic) chemical computer is discussed in “Information Processing Using Networks of Chemical Oscillators” [4]. It is argued that a far-from-equilibrium and oscillatory chemical-reaction system can mimic a small (three “chemical qubits”) network, reminiscent of a certain one-task (chemical) computing device. As an example, the author considers a small network of three coupled chemical oscillators designated to differentiate the white from the red points of the Japan flag. The results are based on computer simulations with the two-variable Oregonator model of the oscillatory Belousov–Zhabotinsky chemical reaction. An optimized network of three interacting oscillators can discriminate between the (red vs. white) colors of randomly selected points with more than 98% accuracy.

Another paper collected by the SI which refers to CL systems, “Effect of Ion and Binding Site on the Conformation of Chosen Glycosaminoglycans at the Albumin Surface” [5], tackles the problem of ion-involving conformation of biopolymers called glycosaminoglycans near certain biopolymeric surfaces. Because of its negative surface charge, albumin plays an essential role in many physiological processes, including the ability to form molecular complexes. In turn, glycosaminoglycans such as hyaluronan and/or chondroitin sulfate are key ingredients of synovial fluid involved in the boundary lubrication regime of an articular cartilage. This study [5] uncovers an impact of relatively small ions (Na^+^, Mg^2+^ and Ca^2+^), on human serum albumin–hyaluronan/chondroitin-sulfate interactions examined by means of molecular docking followed by molecular dynamics simulations. A certain type of glycosaminoglycan binding is analyzed by using a conformational entropy approach, and several protein–polymer complexes are studied to inspect, specifically, how the binding site and presence of ions influence their affinity conditions.

The next CL-type paper [6] of the overarching SI, titled “Does Social Distancing Matter for Infectious Disease Propagation? An SEIR Model and Gompertz Law Based Cellular Automaton”, introduces a stochastic synchronous cellular automaton defined on a square lattice. Its goal is the examination of a population dynamics phenomenon based on certain automaton (Gompertz’s mortality law)-involving rules. The automaton rules are based on the SEIR (susceptible → exposed → infected → recovered) model with probabilistic parameters gathered from real-world data on human mortality and the characteristics of the SARS-CoV-2 disease. With computer simulations, it is shown that there is an appreciable influence of the radius of the neighborhood on the number of infected and deceased individuals in the artificial population. Symptomatically, for a wide range of interactions of exposed individuals (having neither certain hallmarks of the disease nor having been diagnosed by suitable tests), even isolation of infected agents cannot prevent the progress of disease propagation. This supports offensive testing against the disease as one of the meaningful strategies to prevent high peaks of infection in the spread of SARS-CoV-2-like diseases and those similar.

Another paper from the CL collection, “Proof of Equivalence of Carnot Principle to II Law of Thermodynamics and Non-Equivalence to Clausius I and Kelvin Principles” [7], covers a fundamental issue of the laws of thermodynamics. Based mainly on logical rules, the author considers a principal assertion that the Kelvin principle is a weaker statement than the so-called first Clausius principle, and the latter is a weaker statement than the Carnot principle, which is equivalent to the (so-named) second Clausius principle. As a result, the Kelvin principle and the first Clausius principle are not exhaustive formulations of the second law of thermodynamics. It turns out that the Carnot principle eventually becomes such a formulation. The author indicates where the methodological errors were made in the presented proof of the equivalence of the Kelvin principle and both Clausius’ principles mentioned.

The paper titled “Mixedness, Coherence and Entanglement in a Family of Three-Qubit States” [8] regards the problem of a family of states representing three-qubit systems. Formulae are derived, showing the relations between linear entropy and measures of coherence such as degree of coherence by first- and second-order correlation functions. It is demonstrated that qubit–qubit states are strongly entangled when linear entropy approaches some range of values. For such states, the authors derived the conditions determining boundary values of the corresponding entropy as being well-parametrized by the measures of coherence.

The second from the small series of QU papers, “Coherence and Anticoherence Induced by Thermal Fields” [9], addresses coherence and correlations emerging between superpositions of two bosonic modes when the modes are coupled to a third intermediate mode, and are also coupled to external modes, i.e., thermal states of unequal mean photon numbers. It turns out that one of the linear superpositions of the modes, which is efficiently decoupled from the other modes, can express a perfect coherence with the other orthogonal superposition of the modes, and can at the same time exhibit anticoherence with the intermediate mode, giving rise to entanglement between the modes. Such a system can be employed to cool modes to low-temperature values. It is demonstrated that the entanglement between the directly coupled superposition and the intermediate modes may appear in a less restricted range of the number of the thermal photons, such that the modes could be tightly entangled, even at large numbers of the thermal photons.

The last in-presentation-order (QU) paper of the SI “The Generalized Euler Characteristics of the Graphs Split at Vertices” [10] argues that there is a relationship between the generalized Euler characteristic of an original graph that was split at vertices into two disconnected subgraphs, and their generalized Euler characteristics. The theoretical results are experimentally justified by employing microwave networks that mimic quantum graphs. It is shown that the evaluation of the generalized Euler characteristics allows determining the number of vertices for which case the two subgraphs were initially connected.

To summarize, this SI has focused on relevant and fundamental issues of statistical classical/quantum physics (and related subdisciplines), pointing to maximum-entropy and entropy production (and/or the spread of information) principles experienced by the respective CL and QU systems in (non)equilibrium conditions. The studies [1,2,3,4,5,6,7,8,9,10] disclose both the theoretical depth as well as the practical usefulness of the applied CL and QU approaches.
